# Prion strains associated with iatrogenic CJD in French and UK human growth hormone recipients

**DOI:** 10.1186/s40478-021-01247-x

**Published:** 2021-08-28

**Authors:** Jean-Yves Douet, Alvina Huor, Hervé Cassard, Séverine Lugan, Naïma Aron, Chloé Mesic, Didier Vilette, Tomás Barrio, Nathalie Streichenberger, Armand Perret-Liaudet, Marie-Bernadette Delisle, Patrice Péran, Jean-Philippe Deslys, Emmanuel Comoy, Jean-Luc Vilotte, Katayoun Goudarzi, Vincent Béringue, Marcelo A. Barria, Diane L. Ritchie, James W. Ironside, Olivier Andréoletti

**Affiliations:** 1 UMR INRAE ENVT 1225, Interactions Hôtes-Agents Pathogènes, École Nationale Vétérinaire de Toulouse, 23 Chemin des Capelles, 31076 Toulouse, France; 2 BIORAN Team, Lyon Neuroscience Research Center, CNRS UMR 5292 – INSERM U1028, Université de Lyon – Université Claude Bernard, Lyon, France; 3 Neurochemistry Laboratory, Biochemistry Department, Centre de Pathologie et Neuropathologie Est, Hospices Civils de Lyon, Bron, France; 4 Laboratoire Universitaire d’Anatomie Pathologique, INSERM U 1214 TONIC, CHU Purpan, C.H.U. Rangueil - 1 Avenue Jean Poulhès, 50032 - 31059 Toulouse Cedex9, TSA France; 5 CEA, Service d’Etude des Prions et des Infections Atypiques, Université Paris-Saclay, 18 Route du Panorama, 92265 Fontenay-aux-Roses, France; 6 INRAE, AgroParistech, GABI, Université Paris-Saclay, Jouy-en-Josas, France; 7 INRAE, UVSQ, VIM, Université Paris-Saclay, 78350 Jouy-en-Josas, France; 8 National Creutzfeldt-Jakob Disease Research and Surveillance Unit, Centre for Clinical Brain Sciences, Western General Hospital, University of Edinburgh, Edinburgh, EH4 2XU UK

**Keywords:** Prion disease, Iatrogenic CJD, Growth hormone

## Abstract

Treatment with human pituitary-derived growth hormone (hGH) was responsible for a significant proportion of iatrogenic Creutzfeldt–Jakob disease (iCJD) cases. France and the UK experienced the largest case numbers of hGH-iCJD, with 122 and 81 cases respectively. Differences in the frequency of the three *PRNP* codon 129 polymorphisms (MM, MV and VV) and the estimated incubation periods associated with each of these genotypes in the French and the UK hGH-iCJD cohorts led to the suggestion that the prion strains responsible for these two hGH-iCJD cohorts were different. In this study, we characterized the prion strains responsible for hGH-iCJD cases originating from UK (n = 11) and France (n = 11) using human PrP expressing mouse models. The cases included *PRNP* MM, MV and VV genotypes from both countries. UK and French sporadic CJD (sCJD) cases were included as controls. The prion strains identified following inoculation with hGH-iCJD homogenates corresponded to the two most frequently observed sCJD prion strains (M1^CJD^ and V2^CJD^). However, in clear contradiction to the initial hypothesis, the prion strains that were identified in the UK and the French hGH-iCJD cases were not radically different. In the vast majority of the cases originating from both countries, the V2^CJD^ strain or a mixture of M1^CJD^ + V2^CJD^ strains were identified. These data strongly support the contention that the differences in the epidemiological and genetic profiles observed in the UK and France hGH-iCJD cohorts cannot be attributed only to the transmission of different prion strains.

**Supplementary Information:**

The online version contains supplementary material available at 10.1186/s40478-021-01247-x.

## Introduction

Prion diseases (otherwise known as transmissible spongiform encephalopathies) are a group of transmissible neurodegenerative disorders that occur naturally in man and a range of other mammalian species, including scrapie in sheep and goats, and chronic wasting disease in deer and elk. Prion diseases are characterized by the accumulation of a misfolded form of the normal cellular prion protein (PrP^C^) in the central nervous system (CNS) [1]. This misfolded protein (commonly referred to as PrP^Sc^) is considered to be the major, if not the only, component of the transmissible agents or prions that are responsible for these diseases [2]. Serial passage of sheep scrapie in mice by intracerebral inoculation identified the presence of distinct strains of the transmissible agent based on the individual biological properties in the mice that become stable on serial passage [3]. These properties include the disease incubation period and neuropathological phenotype, particularly the distribution and severity of spongiform change in the brain. In the apparent absence of any nucleic acid associated with the transmissible agent, these variations in strain properties have been accounted for by conformational variability in PrP^Sc^ that is self-propagating [2].

Human prion diseases occur in sporadic, genetic and acquired forms. The commonest of these is the sporadic form of Creutzfeldt–Jakob disease (sCJD), which has a worldwide distribution at a remarkably consistent incidence of 1–2 cases per million population per annum. The acquired forms of human prion disease include kuru, iatrogenic Creutzfeldt–Jakob disease (iCJD) and variant Creutzfeldt–Jakob disease (vCJD) [4].

The clinicopathological phenotype of human prion diseases is variable, particularly in sCJD. One major determinant of this variability is the naturally occurring polymorphism at codon 129 in the human prion protein gene (*PRNP*), which can encode either methionine (M) or valine (V), resulting in three possible genotypes: MM, MV and VV. The second major determinant is the isoform of the protease-resistant core of PrP^Sc^ (referred to as PrP^res^) which may be detected by Western blot analysis in brain tissue from affected patients. PrP^res^ occurs as two major isoforms in sCJD cases that differ in the molecular weight of the unglycosylated fragments, designated Type 1 (21 kDa) and Type 2 (19 kDa). Distinct subtypes of sCJD have been recognized according to their clinical and neuropathological features, which largely correspond to the six possible codon 129 genotype/PrP^res^ type combinations (MM1, MV1, VV1, MM2, MV2 and VV2) [5]. However, both type 1 and type 2 PrP^res^ can be detected within either the same or different brain areas in up to 35% of sCJD patients [6, 7]. It has been proposed that the different sCJD subtypes could result from the propagation of different strains of sCJD prions in patients. Experimental transmissions of sCJD brain homogenates to transgenic mice have demonstrated the existence of at least five strains of sCJD prions [8–13].

Although the precise relationship between prion strain diversity and the clinicopathological subtypes of sCJD remains imperfectly characterized, two strains named M1^CJD^ and V2^CJD^ seem to account for the most frequent forms of sCJD; M1^CJD^ is predominantly found in the brain of MM/MV1 sCJD patients, while the V2^CJD^ strain is generally found in VV/MV2 sCJD patients [8, 9, 11, 14]. However, in up to 35% of sCJD patients the co-existence of both M1^CJD^ and V2^CJD^ strains was demonstrated by bioassay [11].

The first reported case of iatrogenic CJD (iCJD), occurring in a recipient of a corneal graft from a donor who had died from sCJD, was published in 1974 [15]. Since then, cases of iCJD have also been identified in small numbers of patients on whom neurosurgical instruments or intracerebral electrodes previously used on the brains of patients with CJD were subsequently used [16]. Larger numbers of iCJD cases (over 200 in each group) have occurred in patients inoculated with human pituitary-derived growth hormone (hGH), and in patients who received an implant of lyophilised human dura mater (hDM) during neurosurgery. Treatment of primary or secondary growth hormone deficiency in children with hGH was commenced initially in the 1950s on a small scale in a few countries. The subsequent clinical success of this treatment resulted its use on a much larger scale and in more countries. The first case of iCJD in a patient treated with hGH was reported in 1985, since when the use of hGH has ceased in many countries and replacement therapy with biosynthetic growth hormone was instigated [16].

The incidence of hGH-iCJD varies from country to country and appears to be related to the scale of the local hGH production process and the likelihood of prion contamination in the selection of autopsy cases for pituitary collection, e.g. the collection of pituitary glands from elderly patients. Since 1985, over 240 cases of iCJD in hGH recipients have been reported in several countries, with the largest numbers of cases occurring in France and the United Kingdom (UK). Four cases of iCJD in human pituitary gonadotrophin recipients have also occurred, all in Australia; one of these patients died in the UK [16].

Overall, 1849 patients from the UK and Ireland were treated with hGH produced in the UK from 1959 until 1985. Since 1985, 81 deaths from iCJD have occurred in this cohort. It has been established that one particular preparation of UK pituitary-derived hGH (the Hartree-modified Wilhelmi (HWP) preparation) had been administered to all hGH recipients who had developed iCJD, albeit from different batches, in varying quantities and over different time periods [17]. In France, 122 cases of iCJD in 1443 hGH recipients have occurred between 1991 and 2019. All were treated between December 1983 and July 1985, which has been identified as a high- risk period for hGH-iCJD in France [18, 19].

Deaths from hGH-iCJD continue to occur in France and in the UK, with the most recent death occurring in 2019 and 2020 respectively, 35 years since hGH therapy ceased in these countries. A precise calculation of incubation periods in human pituitary hormone recipients is difficult, since the patients are often treated over a period of years; the time period from the mid-point of pituitary hormone treatment to the onset of clinical symptoms of iCJD is therefore used as an estimate for the incubation period.

A major factor influencing incubation periods in hGH-iCJD is the *PRNP* codon 129 polymorphism of the recipient. It has long been recognized that homozygosity at this locus may predispose to both iCJD and sporadic CJD (sCJD) [20]. In the healthy UK and French populations, the distribution of the individual genotypes at codon 129 of the *PRNP* gene is similar at around 40% MM, 10% VV and 50% MV [16, 21].

Variations in the frequency of the three *PRNP* codon 129 polymorphisms (MM, MV and VV) and estimated incubation periods associated with each of these three genotypes have been reported in hGH-iCJD cohorts in different countries [16].

In France, the mean incubation period for hGH-iCJD has been estimated at 14.2 years. However, incubation periods differed according to the patient’s age at start of treatment, with the shortest incubation periods observed in the group of patients aged between 10 and 13.7 years when treatment started. Incubation periods were also influenced by the patient’s *PRNP* codon 129 genotype, with mean values of 12.6 years in MM and VV homozygotes and 17.6 years in MV heterozygotes [18].

The risk of developing iCJD in the UK hGH recipients was greatest in those patients who started treatment at ages 8–10 years [17]. Estimated incubation periods in the UK hGH-iCJD patients range from 7 to 40 years; these prolonged incubation periods are reminiscent of those occurring in kuru, where incubation periods of over 40 years have been reported [22]. The incubation periods in the UK hGH-iCJD patients are also influenced by the patient’s *PRNP* codon 129 genotype, but in contrast to the French patients, UK VV homozygous patients had a mean incubation period of 14.3 years, while in MV heterozygous patients the mean incubation period was 23.4 years. The longest incubation periods in UK patients occurred in codon 129 MM homozygotes (mean value 30.8 years) [22].

These observations raised the hypothesis that different prion strains (most likely originating from undetected cases of sCJD in the donor population) contaminated the hGH preparations administered to recipients in the UK and in France, resulting in different incubation periods for each of the *PRNP* codon 129 subgroups between these countries [16, 23]. This study aims to test this hypothesis by performing experimental transmissions of 22 hGH-iCJD brain homogenates from patients with all three codon 129 genotypes from France (11 cases) and the UK (11 cases) into a panel of well characterized human-PrP-expressing transgenic mice (tgHu), following an established methodology that has been used extensively to identify sCJD prion strains in a range of tissue samples from France, the UK and Spain [11, 14]. Sporadic CJD cases originating from France and the UK were also used as controls in this study. This is the first study, to our knowledge, where the strain characteristics of prions in hGH-iCJD cases have been defined and compared to sCJD prion strains.

In clear contradiction to the initial hypothesis, the prion strains that were identified in the UK and the French hGH-iCJD cases were not radically different. In the vast majority of the cases originating from both countries, the V2^CJD^ strain or a mixture of M1^CJD^ + V2^CJD^ strains were identified by the strain typing in tgHu. These data strongly support the contention that differences observed in the epidemiological profiles between hGH-iCJD cases in the UK and France cannot be attributed solely to the transmission of different prion strains.

## Materials and methods

### Ethical statement

All animal experiments were performed in compliance with institutional and French national guidelines in accordance with the European Union Directives 86/609/EEC and 2010/63/EU. Experiments were approved by the Committee on the Ethics of Animal Experiments of the author’s institutions: INRA Toulouse/ENVT (Permit Number: 01734.01).

Concerning the human CJD samples, in all cases informed consent for research was obtained and the material used had appropriate ethical approval for use in this project.

France: human brain samples were obtained from the Brain Bank of CHU of Toulouse, Cardiobiotec (Centre de Resources Biologiques des Hospices Civils de Lyon) and former French national reference laboratory (CEA, Fontenay aux roses) under approval number AC62009-973/20-01-2010. Samples were pseudo-anonymized before dispatch.

UK: Human brain samples were obtained from the National CJD Research and Surveillance Unit Brain and Tissue Bank in Edinburgh, UK, which is part of the MRC Edinburgh Brain Bank. For the purposes of this study, samples were pseudo-anonymized using a Brain Bank reference number. All UK cases had informed consent for research and their supply and use in this study was covered by Ethics Approval (LREC 2000/4/157: National Creutzfeldt–Jakob disease tissue bank: acquisition and use of autopsy material for research on human transmissible spongiform encephalopathies, Professor James Ironside, amended date: 9th October 2007).

### hGH-iCJD cases

22 cases of hGH-iCJD, each of which had frozen tissue (200 to 500 mg) available (cerebral cortex or cerebellar cortex), were included in this study. The patients originated from France and the UK. A diagnosis of iatrogenic CJD was made in each case according to established international criteria (ECDC 2021: https://www.ecdc.europa.eu/en/infectious-diseases-public-health/variant-creutzfeldt-jakob-disease/eu-case-definition). The UK cases are part of a larger series of UK hGH-iCJD cases that has been extensively characterised in terms of neuropathological features, PrP^res^ profiles and prion protein amplification properties [24]. None of the patients had a familial history of prion disease and, in each case, the entire *PRNP* coding sequence was analysed [25, 26]. For each patient the age at death and the estimated incubation period (based on individual hGH treatment histories) are indicated (Table 1).

**Table 1:** Transmission of tissue isolates (10% cerebral/cerebellar cortex homogenates from hGH-iCJD cases) into mice expressing the human PrP (methionine, valine, at codon 129)

Isolate number	Cases					Brain area
	Codon 129	Origin	Year	Age	Incub period	
1	MM	Fr	2001	31	16	Cort
2		Fr	2006	39	21	Cort
3		Fr	2006	36	23	Cort
4		Fr	2003	33	20	Cereb
5		Fr	2004	35	21	Cort
6		UK	2011	46	29	Cort
7		UK	2012	42	32	Cort
8	MV	UK	2001	30	20	Cort
9		UK	1999	37	19	Cort
10		Fr	2006	38	25	Cort
11		Fr	2008	27	24	Cereb
12		Fr	2003	35	23	Cereb
13		UK	1990	34	17	Cort
14	VV	UK	1998	27	16	Cort
15		UK	1997	36	19	Cort
16		UK	1995	33	18	Cort
17		UK	1994	25	11	Cort
18		Fr	1991	11	5	Cort
19		Fr	2000	27	16	Cort
20		UK	1993	30	13	Cort
21		UK	1992	31	16	Cort
22		Fr	1999	24	15	Cort

Isolate number	TgMet_129_						TgVal_129_			
	Passage 1		Passage 2		Passage3		Passage 1		Passage 2	
	Survival time (mean ± SD)	PrP^res^	Survival time (mean ± SD)	PrP^res^	Survival time (mean ± SD)	PrP^res^	Survival time (mean ± SD)	PrP^res^	Survival time (mean ± SD)	PrP^res^
1	585 ± 29	1	487 ± 34	1	ND		173 ± 3	2	171 ± 2	2
2	582 ± 81	1	492 ± 57	1	ND		162 ± 3	2	179 ± 7	2
3	569 ± 76	1	> 400*				163 ± 7	2	181 ± 11	2
4	593 ± 118	1	298 ± 61	1	206 ± 9	1	195 ± 13	2	165 ± 3	2
5	453 ± 137	1	204 ± 4	1	ND		180 ± 6	2	174 ± 9	2
6	246 ± 33	1	213 ± 10	1	ND		206 ± 6	2	161 ± 3	2
7	230 ± 5	1	193 ± 6	1	ND		313 ± 5	1	271 ± 5	1
8	562 ± 55	1	456 ± 6	1	ND		211 ± 12	2	173 ± 7	2
9	635 ± 66	1	503 ± 19	1	ND		172 ± 10	2	177 ± 7	2
10	525 ± 32	1	488 ± 31	1	ND		188 ± 15	2	169 ± 10	2
11	511 ± 29	1	> 400*	1	ND		164 ± 5	2	166 ± 9	2
12	566 ± 170	1	299 ± 42	1	194 ± 4	1	160 ± 10	2	163 ± 4	2
13	337 ± 66	1	212 ± 13	1	ND		193 ± 5	2	176 ± 15	2
14	626 ± 57	1	613 ± 21	1	ND		176 ± 9	2	ND	2
15	423 ± 23	1	669 ± 56	1	ND		180 ± 9	2	175 ± 5	2
16	665 ± 15	1	484 ± 30	1	ND		165 ± 5	2	175 ± 14	2
17	628 ± 83	1	568 ± 82	1	ND		198 ± 7	2	169 ± 7	2
18	565 ± 19	1	> 400*	1			165 ± 5	2	180 ± 10	2
19	759 ± 64	1	> 400*	1			171 ± 7	2	177 ± 8	2
20	612 ± 122	1	312 ± 49	1	202 ± 4	1	178 ± 7	2	180 ± 7	2
21	446 ± 165	1	210 ± 12	1	ND		194 ± 7	2	170 ± 3	2
22	362 ± 48	1	229 ± 8	1	ND		439**	1	ND	

Transgenic mice that express the Met_129_ (tgMet), Val_129_ (tgVal) human PrP were inoculated intra-cerebrally (20µL per mouse) with human growth hormone (hGH) iatrogenic Creutzfeldt–Jakob brain tissue homogenates (frontal, temporal or parietal cortex: (cort) or cerebrellar cortex: (cereb)) from patients originating (orig.) from France (Fr), or the United Kingdom (UK). The hGH-iCJD patients displayed different *PRNP* genotypes at codon 129 (MM: homozygous Met_129_, VV: homozygous Val_129_, MV: heterozygous Met/Val_129_). For each patient, the country of origin, the year of death and age in years at death are indicated. The estimated duration of the incubation period in years (based on the hGH treatment history) is also indicated. After the first and second passages, brain tissue from the clinically affected mice were pooled and used for a next passage in the same line. The PrP^res^ WB isoforms (type 1 or type 2) identified in mouse brains are reported for each two passages. Survival times are shown as mean ± standard deviation (SD). 100% attack rate transmission were observed in all cases

*ND* not done

*Transmission still ongoing with no dead animals observed at the reported date

**A single inoculated animal was positive

### Tissue homogenate preparation

For each hGH-iCJD case, 175 ± 20 mg of frozen brain tissue was homogenized in 5% glucose in distilled water in grinding tubes (Bio-Rad) adjusted to 10% (w/v) using a TeSeE™ Process 48™ homogenizer (Bio-Rad).

### Transgenic mouse lines

Tg340 and tg361 mouse lines that express human PrP methionine at codon 129 or valine at codon 129, respectively, in a prion protein gene knockout (PrP^Ko^) background, were generated as previously described [9, 27]. Both tg340 (tgMet) and tg361 (tgVal) are homozygous for human *PRNP* gene.

### Mouse bioassays

Six- to ten-week-old female mice were anesthetized and inoculated with 2 mg of brain equivalent (20 µL of a 10% cerebral or cerebellar cortex homogenate) in the right parietal lobe using a 25-gauge disposable hypodermic needle.

Mice were observed daily and their neurological status was assessed weekly by qualified veterinarians. Three signs of neurological dysfunction (tremor, ataxia, difficulty righting from supine position, rigidity of tail, kyphosis, paralysis of the lower limbs or bradykinesia,) were necessary to score a mouse positive for prion disease [28].

When clinically progressive TSE disease was evident, the animals were euthanized and their brains harvested. Half of the brain from those animals that had displayed TSE clinical signs was fixed by immersion in 10% formol saline and the other half was frozen at − 20 °C. Tissues from animals found dead were frozen (no formalin fixation). Incubation period was expressed as the mean of the survival (time to death) days post inoculation (dpi) of all the symptomatic mice scored positive for PrP^res^, with its corresponding standard deviation.

### Abnormal PrP western blot (WB) detection

PK resistant abnormal PrP extraction (PrP^res^) and Western blot were performed as previously described [29]. Immunodetection was performed using monoclonal PrP-specific antibodie Sha31 (1 µg/ml), which recognize the amino acid sequence YEDRYYRE (145–152) [30].

### Vacuolar lesion profiles

Hematoxylin–Eosin-stained paraffin-embedded brain tissue sections were used to establish standardized vacuolar lesion profiles in mice as previously described [3, 31]. Each lesion profile was based on data obtained from 4 to 6 animals.

### Reference M1 and V2 CJD strains

Successive 1/10 dilutions of 10% brain homogenate (frontal cortex) from a sCJD MM1 and a sCJD VV2) case were inoculated intra-cerebrally to tgMet (n = 6) and tgVal (n = 6). These data were already presented in a previous study [32] as cases 1 and 6, respectively. The brain of the last PrP^Sc^ positive MM1 in tgMet and VV2 in tgVal mice (in highest dilution groups) were used to re-inoculate groups of tgMet (n = 12) and tgVal (n = 12). The brain of these tgMet and TgVal animals were pooled to constitute two stocks of reference material (named M1^CJD^ and V2^CJD^). The V2^CJD^ stock homogenate was end point titrated by bioassay in tgVal (inoculation of successive 1/10 dilutions of 10% homogenates—Additional file 1: Table S1).

### Data availability

The authors confirm that the data supporting the findings of this study are available within the article and its supplementary material*.*

## Results

22 hGH-iCJD cases were selected, which originated either from France (n = 11) or the UK (n = 11) and included all three possible *PRNP* genotypes at codon 129 (Homozygous Met_129_ or Val_129_, Heterozygous Met_129_/Val_129_) (Table 1). In both the UK and France, the availability of hGH-iCJD cases from certain *PRNP* genotypes (such as MM in the UK or VV in France) with suitable biological (frozen) material was limited. Information on the selected 22 cases, including the estimated incubation period of the disease is included in Table 1.

From the frozen material available, tissue homogenates (10% weight volume) corresponding to either cerebral (frontal, parietal or temporal) cortex or cerebellar cortex from each hGH-iCJD case were transmitted (two iterative passages) to mice homozygous for either methionine (tgMet) or valine (tgVal) at codon 129 of human PrP (Table 1). These two tgHu PrP mouse lines have been shown to express approximately fourfold more PrP^C^ in their brain than human brain tissue [9].

In parallel, French (n = 7) and UK (n = 4) cases of sCJD, classified as MM1, MV1, MV2 and VV2, were transmitted to tgMet and TgVal. (Table 2). Data from these sCJD transmissions have been previously reported in a recent study that aimed to type the prion strains in the brain of MM/MV1 and VV/MV2 sCJD affected patients [11]. Based on the transmission properties of incubation period, lesion profile and biological cloning of prion component present in different brain isolates, this study allowed the identification of two sCJD strains, named as M1^CJD^ and V2^CJD^, that could be present as either pure components or as a mixture in the sCJD isolates (Table 2).

**Table 2:** Transmission of sporadic CJD isolates (10% brain homogenates) into mice expressing the human PrP (methionine, valine, at codon 129)

Isolate number	Codon 129	PrP^res^ type	Origin	Brain area	TgMet_129_				TgVal_129_				Identified strain(s)
					Passage 1		Passage 2		Passage 1		Passage 2		
					Survival time	PrP^res^ type	Survival time	PrP^res^ type	Survival time	PrP^res^ type	Survival time	PrP^res^ type	
23	MM	1	Fr	Frontal cortex	230 ± 13	1	201 ± 11	1	336 ± 8	1	284 ± 5	1	M1^CJD^
24	MM	1	UK	Frontal cortex	226 ± 4	1	ND		326 ± 9		ND		M1^CJD^
25	MV	1	Fr	Frontal cortex	276 ± 19	1	205 ± 7	1	296 ± 13	1	282 ± 9	1	M1^CJD^
26	MV	1	UK	Frontal cortex	197 ± 6	1	209 ± 17	1	289 ± 13	1	289 ± 8	1	M1^CJD^
27	MV	1	Fr	Frontal cortex	205 ± 16	1	220 ± 13	1	258 ± 33	2	184 ± 6	2	M1^CJD^ + V2^CJD^
28	MV	2	UK	Frontal cortex	470 ± 23	1	211 ± 12	1	184 ± 9	2	180 ± 6	2	M1^CJD^ + V2^CJD^
29	MV	2	Fr	Frontal cortex	375 ± 45	1	205 ± 5	1	251 ± 66	2	168 ± 3	2	M1^CJD^ + V2^CJD^
30	MV	2	UK	Frontal cortex	578 ± 28	1	492 ± 70	1	219 ± 17	2	174 ± 3	2	V2^CJD^
31	MV	2	Fr	Caudate	518 ± 84	1	571 ± 14	1	180 ± 8	2	161 ± 8	2	V2^CJD^
32	VV	2	Fr	Frontal cortex	626 ± 85	1	548 ± 24	1	198 ± 7	2	170 ± 7	2	V2^CJD^
33	VV	2	Fr	Frontal cortex	521 ± 65	1	216 ± 1	1	188 ± 13	2	166 ± 6	2	M1^CJD^ + V2^CJD^

Transgenic mice that express the Met_129_ (tgMet), Val_129_ (tgVal) human PrP were inoculated intra-cerebrally (20µL per mouse) with sporadic Creutzfeldt–Jakob (sCJD) brain tissue homogenates (frontal cortex or caudate nucleus) from patients originating (orig.) from France (Fr), or the United Kingdom (UK). The sCJD patients displayed different *PRNP* genotypes at codon 129 (MM: homozygous Met_129_, VV: homozygous Val_129_, MV: heterozygous Met/Val_129_) and PrP^res^ Western blot isoforms (type 1 or type 2). After the first passage, brain tissue from clinically affected mice were pooled and used for a second passage in the same line. The PrP^res^ WB isoforms (type 1 or type 2) identified in mouse brains are reported for each two passages. Survival times are shown as mean ± standard deviation (SD). 100% attack rate transmission were observed in all cases. ND: not done. At the exception of isolate 24, transmission data have already been use in a previous publication. The prion strain(s) identified in each isolate (strain typing based on survival time and vacuolar lesion profile in the brain) are indicated in the table. For full data (lesion profile data and WB PrP^res^ typing) please refer to Cassard et al. [9]

In the French and UK hGH-iCJD isolates, three distinct transmission patterns were observed in mice inoculated with the panel of tissue homogenates.

**Fig. 1:**
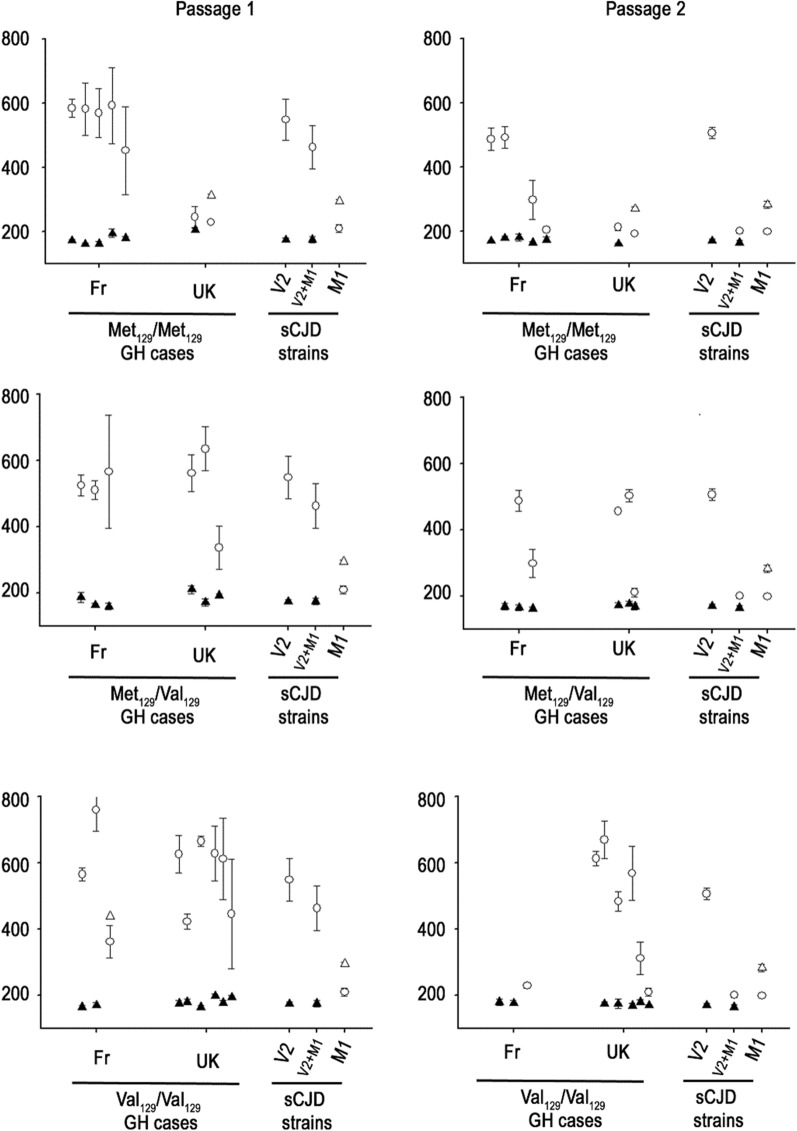
Survival times of human PrP-expressing mice (tgHu) inoculated with growth hormone CJD cases originating from France and UK. Transgenic mice that express the Met_129_ (tgMet), Val_129_ (tgVal) human PrP were inoculated intra-cerebrally (6 mice, 20µL per mouse) with a 10% brain homogenate from hGH-iCJD cases of French and UK origin. Two iterative passages were performed in each mouse line (Table 1). For each passage, results are presented according to the country of origin (Fr and UK) and the *PRNP* codon 129 genotype of patients (homozygous Met129: MM—heterozygous Met129/Val129: MV—homozygous Val129: VV). Survival time (mean ± SD in days post inoculation) in tg Met (○) and tg Val (∆). White/black symbols correspond to a type 1/type 2 PrP^res^ (as assessed by Western blot) in the brain of the mice respectively. Survival times in tgMet and TgVal associated with M1^CJD^ and V2^CJD^ cloned strains as well as an artificial mixture of V2^CJD^ + M1^CJD^ (10^–4^ diluted) are included as reference (see Table 2)

**Fig. 2:**
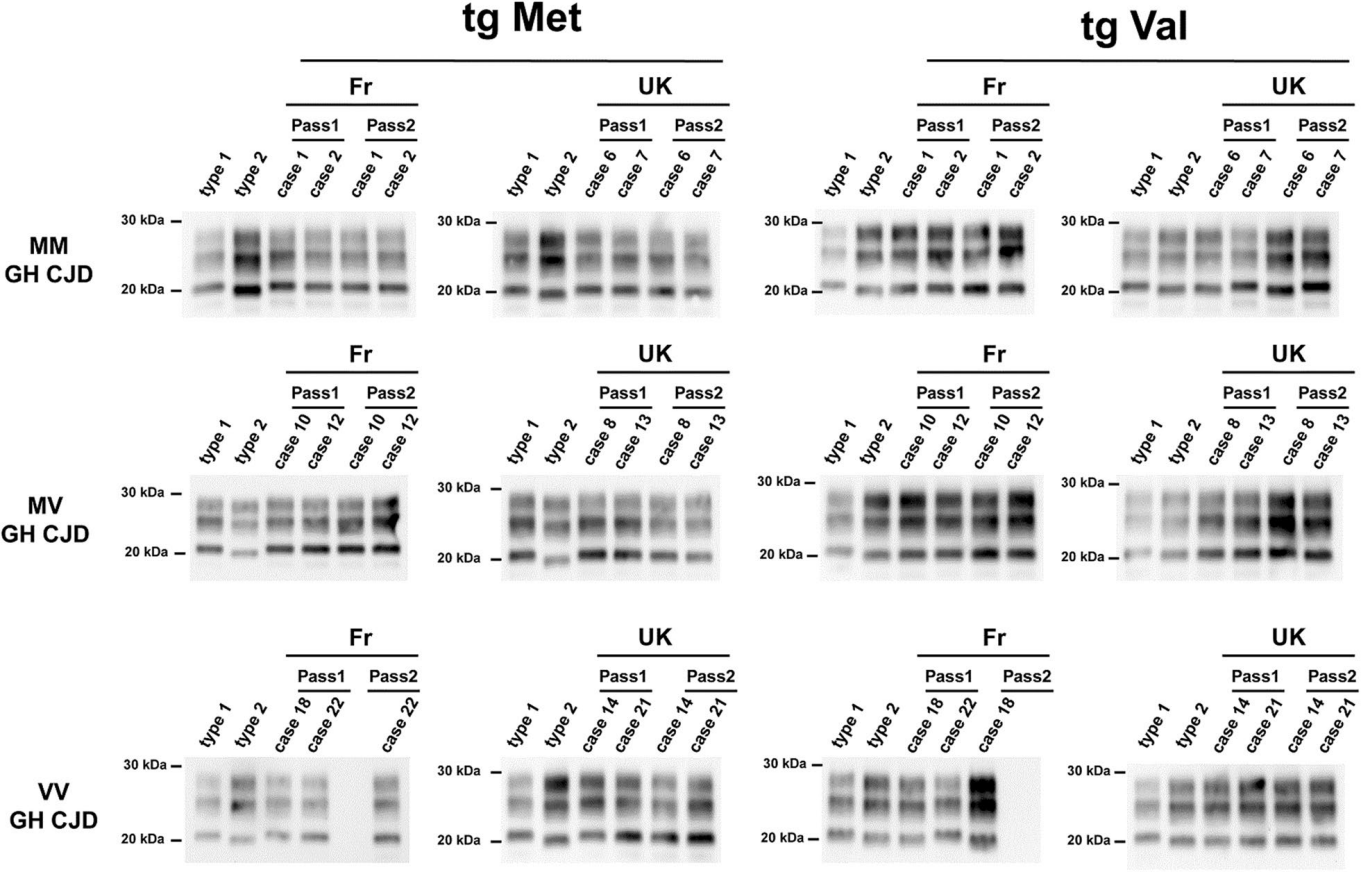
PrP^res^ western blot profiles in the brains of human PrP-expressing mice (TgHu) inoculated with human growth hormone iatrogenic CJD (hGH-iCJD) cases. Transgenic mice that express the Met_129_ (tgMet) or Val_129_ (tgVal) human PrP were inoculated intra-cerebrally (6 mice, 20µL per mouse) with a 10% brain homogenate from hGH- iCJD cases of French and UK origin. Two iterative passages were performed in each line (Table 1). After each passage and in each mouse line the isoform (type 1/type 2) of the PrP^res^ was determined in mice brain by SDS-PAGE and WB with the anti-PrP monoclonal antibody Sha31 (epitope YEDRYYRE). A PrP^res^ type 1 isoform (MM1 sCJD isolate) and type 2 isoform (VV2 sCJD isolate) were included as controls on each gel. The WB results obtain in each line are reported in Table 1

The first and most frequent transmission pattern (n = 13 cases), was observed in French MM (n = 3, cases 1–3), MV (n = 2, cases 10,11) and VV (n = 2, cases 18,19); and UK MV (n = 2, cases 8,9), VV (n = 4, cases 14–17) hGH-iCJD patients. It was characterized by a short incubation period in tgVal (~ 160 to 180 dpi) and a long incubation period in tgMet (~ 400 to 700 dpi) (Table 1, Fig. 1). All tgMet mice accumulated a type 1 PrP^res^ in their brain, while type 2 PrP^res^ was observed in the brain collected from tgVal mice (Table 1, Fig. 2).

**Fig. 3:**
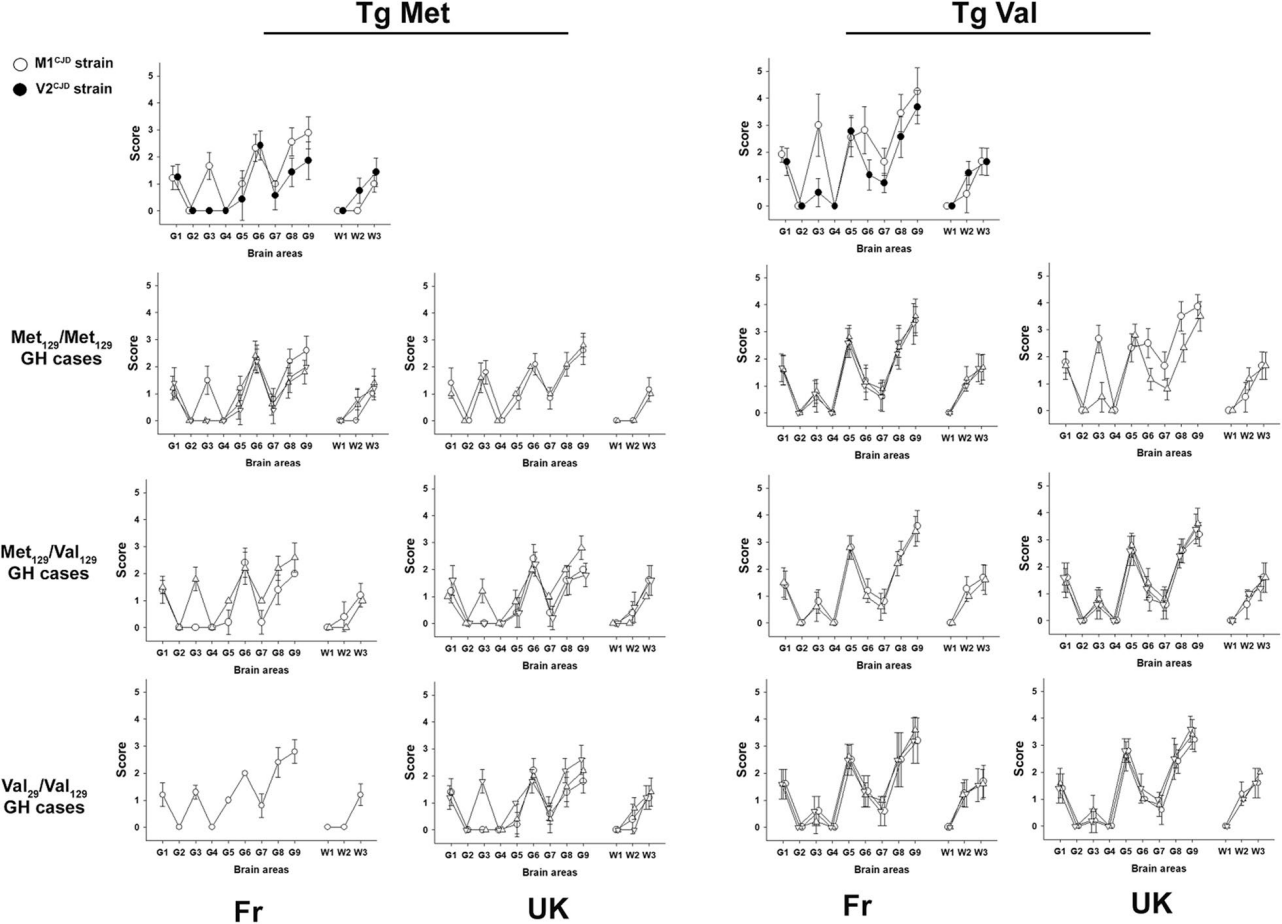
Vacuolar lesion profiles in the brain of human PrP-expressing mice (tgHu) inoculated with human growth hormone iatrogenic CJD cases originating from France and UK. French (Fr) and UK hGH- iCJD isolates were inoculated in transgenic mice that express Met_129_ (tgMet) or Val_129_ (tgVal) human PrP (intra-cerebral route, 6 mice, 20µL per mouse). After two iterative passages standardized vacuolar lesion profiles were established in the brains of the mice. Lesion profiles corresponding to M1^CJD^ (○) and V2^CJD^ (●) strains are presented for comparison. Results are presented according to the origin (Fr and UK) and the genotype of patients (homozygous Met129: MM—heterozygous Met129/Val129: MV—homozygous Val129: VV°). Fr MM cases: case 1 (∇), case 2 (∆), case 5 (○). UK MM: case 6 (○), case 7 (∇). Fr MV: case 10 (○), case 12 (∆). UK MV: case 11: case 9 (∇), cases 9 (○), case 13(∆). Fr VV cases: case 18 (∆), case 19 (∇), case 22 (○). UK VV cases: case 14 (○), case 15 (∆), case 22 (∇). For the Fr VV cases 18 and 19, second passage in tgMet were not available at the moment of writing (see Table1)

This pattern was very similar to that observed in tgMet and tgVal inoculated with the V2^CJD^ strain (Table 3, Fig. 1) which was observed in the brain of some French and UK MV2 and VV2 sCJD-affected patients (cases 30–32) (Table 2). The vacuolar lesion profile in the brain of the mice (after second passage) confirmed the propagation of the V2^CJD^ prion strain in both the tgMet and tgVal that had been inoculated with these 13 hGH-iCJD cases (Fig. 3).

**Table 3:** Bioassay transmission in tg Hu mice of artificial V2^CJD^/M1^CJD^ strains mixture

Artificial strain mixture composition		TgMet_129_						TgVal_129_					
		Passage 1			Passage 2			Passage 1			Passage 2		
		n/no	Survival time (mean ± SD)	PrP^res^ type	n/no	Survival time (mean ± SD)	PrP^res^ type	n/no	Survival time (mean ± SD)	PrP^res^ type	n/no	Survival time (mean ± SD)	PrP^res^ type
V2 neat	M1 neat	6/6	207 ± 3	1	6/6	205 ± 2	1	6/6	173 ± 7	2	6/6	179 ± 1	2
V2 neat	–	6/6	549 ± 65	1	6/6	506 ± 17	1	6/6	175 ± 5	2	6/6	174 ± 3	2
M1 neat	–	6/6	210 ± 12	1	6/6	199 ± 3	1	6/6	295 ± 5	1	6/6	283 ± 11	1
V2 neat	M1 10^–1^	6/6	244 ± 11	1	6/6	203 ± 8	1	6/6	177 ± 6	2		ND	
V2 neat	M1 10^–2^	6/6	250 ± 5	1	6/6	198 ± 3	1	6/6	171 ± 6	2		ND	
V2 neat	M1 10^–3^	6/6	329 ± 89	1	6/6	207 ± 5	1	6/6	173 ± 10	2		ND	
V2 neat	M1 10^–4^	6/6	463 ± 67	1	6/6	201 ± 7	1	6/6	176 ± 10	2		ND	
V2 neat	M1 10^–5^	6/6	513 ± 32	1	6/6	505 ± 24	1	6/6	176 ± 5	2		ND	
M1 neat	V2 10^–1^	6/6	207 ± 10	1		ND		6/6	190 ± 8	2	6/6	179 ± 3	2
M1 neat	V2 10^–2^	6/6	211 ± 9	1		ND		6/6	233 ± 13	2	6/6	189 ± 8	2
M1 neat	V2 10^–3^	6/6	199 ± 9	1		ND		6/6	245 ± 2	2	6/6	189 ± 8	2
M1 neat	V2 10^–4^	6/6	203 ± 8	1		ND		6/6	288 ± 2	1	6/6	229* 266, 267, 287, 288,299	2 1
M1 neat	V2 10^–5^	6/6	202 ± 8	1		ND		6/6	283 ± 8	1	6/6	289 ± 5	1

M1^CJD^ and V2^CJD^ strains were obtained by the endpoint titration of a MM1 and VV2 sCJD isolate in Met_129_ (tgMet) or Val_129_ (tgVal) human PrP-expressing mice respectively (see Additional file 1: Table S1). Brains from tgMet (inoculated with M1^CJD^ strain) and tgVal (inoculated with V2^CJD^ strain) were used to produce stock solutions (10% tissue homogenates). 1/10 dilution series (in phosphate buffer saline) of each stock solution were prepared. M1^CJD^/V2^CJD^ strain mixtures were obtained by mixing equal volume of each component at the chosen dilutions. Samples were then transmitted (two iterative passages) to tgMet and tgVal (intra-cerebral route, 20µL per mouse). Brains from first passage positive mice (PrP^res^ presence in the brain) were pooled and used for the second passage. The PrP^res^ WB isoforms (type 1 or type 2) identified in mouse brains are reported. Survival times (time to death in days) are shown as mean ± standard deviation (SD). n/n0: number of diseased/number of inoculated mice. *PrP^res^ WB profile of the mice in the group were not homogenous and the individual incubation periods of each animal are presented. ND: not done. These data were already used in a previously published study [9]

The second transmission pattern observed was observed in one MM UK hGH-iCJD case (case 7). It was characterized by a short incubation period in tgMet (~ 200 dpi), and a longer one in tgVal (~ 300 dpi) (Table 1, Fig. 1). Irrespective of the inoculated mouse line, a type 1 PrP^res^ was observed in the brain of the inoculated animals (Table 1, Fig. 1). These features were similar to those associated with the transmission of the M1^CJD^ strain (Table 3) which could be observed in French and UK MM1 and in a part of the MV1 sCJD affected patients (cases 23–26) (Table 2). The vacuolar lesion profile in the brain of the inoculated mice (after second passage) confirmed the propagation of M1^CJD^ prion strain in tgMet and tgVal inoculated with hGH-iCJD case 7.

In another case (case 22, French origin, VV genotype), available transmission results and lesion profiles in tgMet and tgVal, were also compatible with the presence of M1^CJD^ strain. However, incomplete second passage of this isolate in tgVal at the time of writing precluded drawing a definitive conclusion on the nature of the prion strain.

A third transmission profile was identified in French MM (n = 2, cases 4, 5) and MV (n = 1, case 12) cases as well as in UK MM (n = 1, case 6), MV (n = 1, case 13) and VV (n = 2, cases 20, 21) patients.

This transmission pattern was characterized on second passage by a short incubation period in tgMet (~ 200–300 dpi), and a short incubation period in tgVal (about 160–200 dpi) (Table 1, Fig. 1). A type 1 PrP^res^ accumulated in the brain of tgMet while type 2 PrP^res^ was observed in tgVal mice (Fig. 2). In these isolates, the vacuolar lesion profiles in the TgMet matched with the profiles observed in mice inoculated with M1^CJD^ strain while in the tgVal mice it corresponded to the V2^CJD^ strain (Fig. 3). Strikingly, the transmission of artificial mixtures containing V2^CJD^ cloned strain and a low dilution (10^–3^ to 10^–4^) of the M1^CJD^ cloned strain in tgMet and TgVal mice resulted in similar transmission patterns (Table 3, Fig. 3).

## Discussion

### Representativeness of the panel of hGH-iCJD cases

hGH-iCJD cases are considered to be the consequence of childhood treatment with human growth hormone, prepared using pituitary glands collected from donors that were either affected with or at a late stage of incubation of sCJD. France (n = 122) and the UK (n = 81) account for the largest numbers of hGH-iCJD patients identified worldwide. The epidemiological profile of the hGH-iCJD cases in these countries differs in the distribution of *PRNP* genotypes at codon 129 and in the mean incubation period in each genotype group. These differences between the UK and France have been proposed to have resulted from contamination with different prion strains [16, 23, 24].

Bioassays in reporter animal models, followed by the phenotyping of the propagated prions (vacuolar lesion profile in the brain and incubation period) remains the gold standard approach for the characterization of prion strains. Over the last decade, transgenic mice that express different human PrP variants at codon 129 (tgHu) have been shown to be valuable models in which to discriminate between different human prion agents and have provided valuable insights in the diversity of the prion strains responsible for sCJD [8–13]. These mouse models have allowed the identification of five different prion strains associated with sCJD among which two, named M1^CJD^ and V2^CJD^, are thought responsible for the most frequent clinico-pathological forms of sCJD (observed in MM/MV1 and VV/MV2 patients, respectively) [8–11]. These same animal models also demonstrated that in around 30% of the MV1, MV2 and VV2 sCJD patients both M1^CJD^ and V2^CJD^ prions were present as a mixture [11].

Transmission of the 11 French and 11 UK hGH-iCJD cases in mice expressing valine 129 (tgVal) and methionine 129 (tgMet) human PrP variants in this study, represents an unprecedented effort to document the nature of the prion strains responsible for this iatrogenic form of the disease. Our study was primarily designed to test the hypothesis that different prion agents were responsible for the hGH-iCJD outbreaks in France and in the UK. While the design of the experiments was fit for this purpose, the materials (cost and duration of bioassay, availability of material from patients) and ethical considerations limited the number of hGH-iCJD cases that could undergo strain typing by bioassay.

### Prion strains in the UK and French hGH-iCJD cases

The prion strains that were identified in brain isolates prepared from French and UK hGH-iCJD cases following bioassay in tgHu mice models correspond to prion strains that were previously identified in sCJD patients in France and in the UK using the same mouse models [11].

In contradiction to the initial hypothesis, the prion strains that were identified in the UK and the French GH-cases were not radically different.

In the vast majority of the UK (10 out 11) and French (10 out 11) cases, the V2^CJD^ strain or a mixture of M1^CJD^ + V2^CJD^ strains were identified by the strain typing bioassays.

A pure V2^CJD^ strain was observed in all three *PRNP* genotypes in French hGH-iCJD patients and in two *PRNP* genotypes (MV and VV) in the UK patients. In both countries, M1^CJD^ + V2^CJD^ strain mixtures were identified in the three different *PRNP* genotypes. A pure M1^CJD^ strain was identified in a single UK patient (case 7, MM genotype) and suspected in a French patient (case 22, VV genotype -definitive transmission results not yet available).

These data strongly support the contention that the difference of epidemiological profile observed between the UK and the French hGH-iCJD outbreaks cannot be attributed to the transmission of different prion strains.

### V2 dominant presence is concordant with previous investigations in the UK

In the UK, several studies have addressed the question surrounding the prion strains responsible for hGH-iCJD, by the characterisation of the neuropathology features and/or the PrP^res^ biochemical properties in the brain of hGH-iCJD affected individuals, including those cases investigated by bioassay in this study [22, 24, 33]. So far, no comprehensive description of the neuropathological and biochemical PrP^res^ properties in the French hGH-iCJD cohort is available in the literature.

In a large number of VV and MV UK hGH-iCJD patients, neuropathological features that are considered characteristic of VV2 sCJD cases (including plaque-like deposits) were identified. Along with the biochemical profile of the UK VV and MV hGH-iCJD cases, this leads to the proposal that pituitary glands collected from VV2 sCJD case(s) might have played a preponderant role in the UK hGH-iCJD outbreak [22, 24].

The results of this study supports this hypothesis, with the unambiguous identification of the V2^CJD^ strain that was obtained by bioassay of brain homogenate from 6 out of the 9 VV and MV UK hGH-iCJD cases.

Similarly, the identification of MM1-like sCJD neuropathological, biochemical and protein misfolding cyclical amplification (PMCA) features reported in case hGH-iCJD 21 in the earlier study by Ritchie et al. concurs with the pure M1^CJD^ strain phenotype we observed in tgHu mice inoculated with cerebral cortex homogenate from the same patient (case 7 in this study). The other UK *PRNP* codon 129 MM hGH-iCJD case reported by Ritchie et al. (case hGH-iCJD 20) had atypical neuropathological and biochemical features along with PMCA features that suggested the influence of a V2 strain. This case was also included in the present study as case 6, which had transmission characteristics different from case 7, suggesting a mixed M1^CJD^/V2 ^CJD^ strain effect. The transmission findings in cases 6 and 7 therefore provide a direct in vivo correlation that confirms the in vitro findings of Ritchie et al. [24].

### Coexistence of several strains in French and UK hGH-iCJD cohorts

The identification of mixed M1^CJD^ and V2^CJD^ strains in both the UK and the French hGH-iCJD cases raises the question of the origin of this strain diversity.

The pooling of pituitary glands during hGH production from several individuals affected with different strains of sCJD may be one explanation for this phenomenon. However, since the co-existence of M1^CJD^ and V2^CJD^ strains have been reported in up to 35% of sCJD cases, the use of pituitary gland from a single donor may also explain the presence of the two strains [7, 11, 34].

In France, 12 batches of extracted hGH, all produced between January 1982 and December 1985, were identified as the potential source of infection. According to available data, a batch was in average produced from 1000 to 1500 pituitary glands, meaning that tissue from up to 18 000 individuals may have contributed to the at-risk batches [18].

In the UK it is estimated that around 200,000 pituitary glands were used to produce the hGH extracts that were used to treat patients that later developed hGH-iCJD [17].

The sCJD prevalence in industrialized countries is estimated to be approximatively 1.5 cases per million people and per year [35]. In people aged over 50, sCJD frequency significantly increases to reach 5.5 to 7.5 case per million people per year in individuals aged between 65 and 79 [36, 37]. However, in France and in the UK (each with a similar population size), between 600,000 and 700,000 deaths are recorded each year, with 80–130 sCJD cases diagnosed annually. Based on these figures, the possibility exists that more than one infectious pituitary gland could have entered in the production of the at-risk hGH batches in both France and the UK.

### Epidemiological profiles in both countries

Assuming that the same prion strains are responsible for the UK and French hGH-iCJD cases, the question remains of how the epidemiological differences occurring between the two hGH-iCJD case cohorts may be explained.

The frequency of Met/Met_129_, Met/Val_129_ and Val/Val_129_ individuals in the general population and in their distribution in sCJD cases are similar in France and in the UK [16]. The hGH therapeutic scheme in both countries, including the route of administration (in general intramuscular) and the average age of administration were also similar, making these parameters unlikely explanations for the observed epidemiological differences [17–19, 23].

In experimental TSE models, longer and more widespread incubation periods are classically observed when animals are exposed to decreasing doses of infectivity [38]. The average incubation duration for hGH-iCJD patients from each *PRNP* codon 129 genotype were significantly longer in the UK patients (VV: 14.3 years–MV: 23.4 years–MM: 30.8 years) when compared with French patients (VV: 9 years–MV: 17.6 years–MM: 12 years) [16, 22]. These elements support the hypothesis that French hGH-iCJD affected patients could have been exposed to higher infectious doses than the UK patients.

Although the relevance of findings in experimental TSE models to the physiopathology of CJD in humans is uncertain, they could provide some valuable insights on the relative abilities of sCJD strains to propagate in hosts expressing different human PrP variants.

The end point titrations of V2^CJD^ and M1^CJD^ in tgMet and tgVal indicated that the M1^CJD^ strain displays a 1000-fold lower capacity to propagate in Val_129_ than in Met_129_ PrP-expressing host (Additional file 1: Table S1). Conversely, one infectious dose (ID_50_) of V2^CJD^ strain (as measured by the intracerebral route in tgVal) transmits with shorter incubation period in tgVal than 10^7.2^ ID_50_ M1^CJD^ strain (as measured by the intracerebral route in tgMet) [11, 32].

When tgHu mice are co-infected with M1^CJD^ and V2 ^CJD^ prions, the nature of the strain(s) that propagate in the brain of the recipients depends on the *PRNP* genotype at codon 129 and to the specific amount of M1^CJD^ and V2^CJD^ infectivity in the inoculum, resulting in the presence of either M1^CJD^ + V2^CJD^ strain mixture or of a pure M1^CJD^ or V2^CJD^ strain in the infected host brain (Table 3) [11]. Based on these elements, a scenario where M1^CJD^ and V2^CJD^ would display different abilities to propagate in an individual according to his genotype at codon 129 and the infectious titre of each strain seems plausible. At low infectious doses, as seems possible in the UK hGH situation, the V2^CJD^ strain would dominate, mostly in VV individuals.

The isolation of a sub-dominant M1^CJD^ strain in homozygous (cases 4 and 5) and heterozygous (case 12) Met_129_ patients indicates that French hGH-iCJD patients were exposed to this prion strain. The transmission of the M1^CJD^ and V2^CJD^ strain in tgMet mice support the view that the M1^CJD^ strain, even if present at very low level in the extractive hGH, should propagate as the dominant strain in the homozygous Met_129_ patients. The identification of the V2^CJD^ strain as dominant prion strain in the brain of the homozygous Met_129_ French hGH-iCJD patients is therefore surprising.

These observations could be explained by the effects of a peripheral (non-CNS) route of transmission that would differentially impact on the relative efficiency of M1^CJD^ and V2^CJD^ transmission by the intramuscular route of exposure in hGH recipients, resulting in a slow or inefficient transmission of the M1^CJD^ strain.

In a given host, the inoculation by a peripheral route of two prion strains can result in radically different transmission profile, depending on their capacity to replicate early in the lymphoid tissues before neuroinvasion. The inoculation of the HY prion strain in hamsters by the intracerebral, the intraperitoneal or the oral route resulted in all three instances in a highly efficient transmission (100% attack rate) of the disease. The inoculation of the DY prion strain in hamsters by the intra cerebral route also resulted in a prion disease. In sharp contrast, the inoculation of a high infectious dose of DY prions in hamsters by the intraperitoneal or the oral route failed to transmit the disease [39, 40]. HY but not DY are able to replicate at detectable levels in the lymphoid tissue. Interestingly, V2^CJD^ strains appeared to replicate at higher levels than M1^CJD^ strain in the lymphoid tissue of tgMet mice [10].

Further investigations will be necessary to confirm the influence of the intramuscular route of infection in modulating M1^CJD^ and V2^CJD^ strain transmission efficiencies. Beyond this, the characterization of the PrP^res^ and measurement of the infectivity levels in the pituitary glands of sCJD patients and the impact of hGH extraction processes on M1^CJD^ and V2^CJD^ strains from the pituitary glands (removal of infectivity) would be very helpful for appreciating the potential exposure conditions to prions in hGH-treated patients. Ultimately, the direct characterisation of prion infectivity levels and strains in the French and UK hGH-batches that appear to have resulted in the transmission of hGH-iCJD would be very informative.

Although numerous elements are still lacking to fully understand the difference existing between the French and UK hGH-iCJD case cohorts, the results that we report in this study indicate that the nature of the prion strains involved are not fundamental to the observed differences in epidemiological profiles.

Finally, the number of hGH-iCJD cases that we investigated (n = 22) should be considered as relatively limited when compared to the total number of hGH-iCJD cases identified in France (n = 122) or the UK (n = 81). Therefore, we cannot be certain that the panel of hGH-iCJD cases that we investigated necessarily provides a full picture of the prion strain diversity in the French and the UK hGH-iCJD cohorts.

## Supplementary Information

**Additional file 1: Table S1**. End point titration of sporadic CJD MM1 and VV2 isolates in transgenic mice expressing the human PrP.

## Abbreviations

hGH: Human pituitary-derived growth hormone
hDM: Human dura mater
iCJD: Iatrogenic Creutzfeldt–Jakob disease
M_129_: Methionine at codon 129 of *PRNP*
tgHu: Human-PrP-expressing transgenic mice
tgMet or tg 340: Homozygous human-PrP-expressing transgenic mice (Methionine 129 variant)
tgVal of tg361: Homozygous human-PrP-expressing transgenic mice (Valine 129 variant)
sCJD: Sporadic form of Creutzfeldt–Jakob disease
V_129_: Valine at codon 129 of *PRNP*
vCJD: Variant Creutzfeldt–Jakob disease
