# Supplementary material for: Prion strains associated with iatrogenic CJD in French and UK human growth hormone recipients
Source: Acta Neuropathol Commun. 2021 Aug 28;9:145. doi: 10.1186/s40478-021-01247-x (PMC8403347; doi:10.1186/s40478-021-01247-x)
Supplement: Supplementary file 1 — Additional file 1: Table S1. End point titration of sporadic CJD MM1 and VV2 isolates in transgenic mice expressing the human PrP. [file 40478_2021_1247_MOESM1_ESM.docx]

**Sup Table 1:** End point titration of Sporadic CJD MM1 and VV2 isolates in transgenic mice expressing the human PrP

|  | **MM1 (case 1)** | | | | |  | **VV2 (case 2)** | | | | |
| --- | --- | --- | --- | --- | --- | --- | --- | --- | --- | --- | --- |
|  | **Tg Met_129_** | |  | **Tg Val_129_** | |  | **Tg Met_129_** | |  | **Tg Val_129_** | |
|  | **Positive mice** | **Incubation period** |  | **Positive mice** | **Incubation period** |  | **Positive mice** | **Incubation period** |  | **positive mice** | **Incubation period** |
| **neat** | 6/6 | 186±10 |  | 6/6 | 286±18 |  | 6/6 | 585±24 |  | 6/6 | 166±11 |
| **10^-1^** | 6/6 | 213±15 |  | 6/6 | 347±16 |  | ND |  |  | 6/6 | 191±9 |
| **10^-2^** | 6/6 | 240±13 |  | 1/6 | 390 |  | ND | - |  | 6/6 | 203±9 |
| **10^-3^** | 6/6 | 263±24 |  | 0/6 | >650 |  | ND | - |  | 6/6 | 252±16 |
| **10^-4-^** | 6/6 | 296±26 |  | 0/6 | >650 |  | ND | - |  | 1/6 | 294 |
| **10^-5^** | 6/6 | 323±29 |  | 0/6 | >650 |  | ND | - |  | 0/6 | >650 |
| **10^-6^** | 1/6 | 316 |  | 0/6 | >650 |  | ND | - |  | 0/6 | >650 |
| **10^-7^** | 0/6 | >600 |  | ND | - |  | ND | - |  | 0/6 | >650 |

Successive 1/10 dilutions of 10% brain homogenate (frontal cortex) from an MM1 and a VV2 sCJD-affected patient were inoculated intracerebrally to tgMet (*n*=6) and tgVal mice (*n*=6). Mice were euthanized when they showed clinical signs of infection or after 650 days. Mice were considered infected when PrP^res^ deposition was detected in their brain by western blot using the Sha31 monoclonal antibody, which recognizes amino acids 145–152 (YEDRYYRE) of the sheep PrP. ND: not done. Incubation periods (in days) are shown as mean±standard deviation (SD) except when less than 100% of the animals developed clinical sign. In that case individual incubation period are presented. The data included in this table were already used in Huor et al 2017 ^16^.
